# Identification of key hub genes and potential therapeutic drugs for nasopharyngeal carcinoma: Insights into molecular mechanisms and treatment strategies

**DOI:** 10.1016/j.bjorl.2025.101618

**Published:** 2025-04-25

**Authors:** Haiyan Quan, Hongguo Yin, Zhen Wang, Yuan Lv, Qiong Sun, Ting Yin

**Affiliations:** a Hunan Polytechnic of Environment and Biology, Hengyang, Hunan, China; b Department of Ophthalmology, The Second Affiliated Hospital, University of South China, Hengyang, Hunan, China

**Keywords:** Nasopharyngeal carcinoma, Key genes, Drug screening, Calcitriol

## Abstract

**Objective:**

Nasopharyngeal Carcinoma (NPC) is a highly malignant cancer with a high incidence in East and Southeast Asia, including southern China. Despite advances in treatment, the prognosis for advanced NPC remains poor due to high recurrence and metastasis rates. The molecular mechanisms driving NPC progression are not fully understood, and identifying key genes and potential therapeutic agents is critical. This study aims to uncover critical genes and screen therapeutic drugs, providing insights into NPC pathogenesis and novel treatment strategies.

**Methods:**

Three GEO datasets (GSE12452, GSE53819, and GSE61218) were analyzed to identify overlapping Differentially Expressed Genes (DEGs) in NPC. Gene Ontology (GO), Kyoto Encyclopedia of Genes and Genomes (KEGG), and Gene Set Enrichment Analysis (GSEA) were used to explore the biological roles of DEGs. Protein-Protein Interaction (PPI) and mRNA-miRNA-lncRNA interaction networks were constructed to identify key hub genes. Potential therapeutic drugs were predicted via a Drug-Gene Interaction network. The overexpression of hub genes was validated in NPC cells using CCK-8 assays, and the anti-proliferative effects of three drugs ‒ valproic acid, cyclosporine, and calcitriol ‒ were tested.

**Results:**

Eight hub genes (ASPM, BIRC5, BUB1B, CDK1, KIF23, PBK, TOP2A, and TTK) were identified, with ASPM reported for the first time in the context of NPC. Overexpression of these genes significantly promoted NPC cell proliferation. Among the tested drugs, calcitriol exhibited the most potent anti-proliferative effect, with IC50 values of 0.90 μM, 0.47 μM, and 0.31 μM at 24-, 48-, and 72-hs, respectively.

**Conclusion:**

This study identified eight key genes as potential biomarkers for NPC and validated calcitriol as a promising therapeutic agent, providing a foundation for further research into NPC treatment.

**Level of evidence:**

Level 2 (Individual cross-sectional studies or systematic review of surveys that allow matching to local circumstances).

## Highlights

• Eight key hub genes identified as drivers of NPC progression.
• Drug screening identified calcitriol as a potent inhibitor of NPC proliferation.
• Provides novel molecular insights and potential therapeutic strategies for NPC.

## Introduction

Nasopharyngeal Carcinoma (NPC) is a malignant tumor originating in the epithelial cells of the nasopharynx, located in the upper throat behind the nose.^1^ While NPC is relatively rare globally, it is highly prevalent in specific regions, particularly Southeast Asia and southern China. The disease is closely linked to Epstein-Barr Virus (EBV) infection, genetic predisposition, and environmental factors such as dietary habits.^2^

EBV plays a crucial oncogenic role in NPC through viral proteins like LMP1, LMP2, and EBNA1, which activate key signaling pathways, including NF-κB, PI3K/AKT, and JAK/STAT.^3^ These pathways promote tumor proliferation, resistance to apoptosis, and immune evasion. However, targeting these viral oncoproteins remains challenging due to their intracellular localization, functional redundancy, and potential off-target effects.^4^, ^5^ While EBV-directed immunotherapies, such as cytotoxic T-cell therapy and therapeutic vaccines, have been explored, their clinical efficacy remains limited, necessitating alternative therapeutic strategies.^6^, ^7^

Genome-Wide Association Studies (GWAS) have identified host susceptibility genes implicated in NPC, including CDKN2A/2B, TNFRSF19, ITGA9, and TERT. CDKN2A/2B (p16, p15) regulate the cell cycle, and their inactivation leads to uncontrolled proliferation.^8^ TNFRSF19 and ITGA9 contribute to tumor invasion,^9^, ^10^ while TERT enhances telomerase activity, promoting tumorigenesis.^11^ Despite their significance, these genes have proven difficult to target due to the complexity of reactivating tumor suppressors or inhibiting broadly expressed signaling molecules.^12^, ^13^

Given the limitations of current therapeutic targets, there is an urgent need to identify novel molecular drivers of NPC. In this context, our study integrates bioinformatics analysis with experimental validation to identify key molecular drivers of NPC and explore their therapeutic implications. Using three GEO datasets, we conducted functional enrichment analysis (GO, KEGG, GSEA), PPI network construction, and a ceRNA regulatory network analysis. We then experimentally validated the role of a hub gene in NPC progression and further screened for potential therapeutic agents targeting this gene using a drug-gene interaction network. Our findings provide new insights into the molecular underpinnings of NPC and highlight a potential therapeutic agent, offering a foundation for future translational research in NPC treatment.

## Methods

### Data acquisition and identification of common differentially expressed genes

Transcriptome sequencing datasets (GSE1245220,^14^ GSE5381921,^15^ and GSE6121822^16^) containing nasopharyngeal carcinoma and normal nasopharyngeal tissues were obtained from the GEO database. Differentially Expressed Genes (DEGs) were identified using the GEO2R online tool, with selection criteria of adjusted *p* < 0.05 and |log₂FC| ≥1. Common DEGs among the three datasets were visualized using a Venn diagram.

### Functional and pathway enrichment analysis

To explore the biological roles of common DEGs, Gene Ontology (GO) annotation, including Biological Process (BP), Cellular Component (CC), Molecular Function (MF) and Kyoto Encyclopedia of Genes and Genomes (KEGG) pathway analysis were conducted using the clusterProfiler package^17^ in R. A *p* < 0.05 was considered statistically significant.

### Gene Set Enrichment Analysis (GSEA)

GSEA was conducted to assess functional enrichment of gene sets based on genome-wide expression profiling.^18^ Genes were ranked by mean expression across datasets, and enrichment analysis was performed using the clusterProfiler package. Significant pathways were identified using adjusted *p*-value < 0.05, False Discovery Rate (FDR) < 0.1, and Normalized Enrichment Score (NES) > 1.6.

### Protein-Protein Interaction (PPI) network and hub gene identification

A PPI network was constructed using the STRING database^19^ with a confidence score ≥0.7 and visualized in Cytoscape (v3.7.2).^20^ The MCODE plug-in was used to identify significant clustering modules (nodes > 10, MCODE score > 8). Hub genes were selected based on node degree using the CytoHubba plug-in.

### Validation of hub genes

HK1 cells were seeded into 96-well plates at a density of 1000 cells per well and incubated overnight. Plasmids were transfected using Lipofectamine 3000 according to the manufacturer’s protocol. After 48 h, cell proliferation was assessed using the CCK-8 assay. At 24-, 48-, 72- and 96-hs post-transfection, 10 μL of CCK-8 reagent was added to each well and incubated for 1 h at 37 °C. Absorbance at 450 nm was measured using a microplate reader. Proliferation rates were compared between the overexpression and control (empty vector) groups.

### Construction of the mRNA-miRNA-lncRNA interaction network

The competing endogenous RNA (ceRNA) network was constructed as follows: (1) miRNA targets of hub mRNAs were predicted using TarBase v.8^21^; (2) Cytoscape and CytoHubba were used to establish the mRNA-miRNA network; (3) lncRNA targets of these miRNAs were predicted using LncBase Predicted v.2;^22^ (4) Differentially expressed lncRNAs (DElncRNAs) were identified from GSE95166^23^ and GSE126683;^24^ (5) Overlapping DElncRNAs were integrated into the ceRNA network.

### Drug-gene interaction network construction

The Comparative Toxicogenomics Database (CTD^25^ was used to identify potential drug-gene interactions. The interaction network was visualized in Cytoscape, where red arrows and green T-lines indicated gene activation and inhibition, respectively.

### Screening and evaluation of potential therapeutic drugs

Candidate drugs not previously reported in NPC were screened using the CCK-8 assay. HK1 cells were treated with different compounds, and viability was assessed at 24-, 48- and 72-hs post-treatment by measuring absorbance at 450 nm. The effects of each drug on cell proliferation were analyzed to identify promising therapeutic agents.

## Results

### Identification of common DEGs in NPC from multiple datasets

**Fig. 1:**
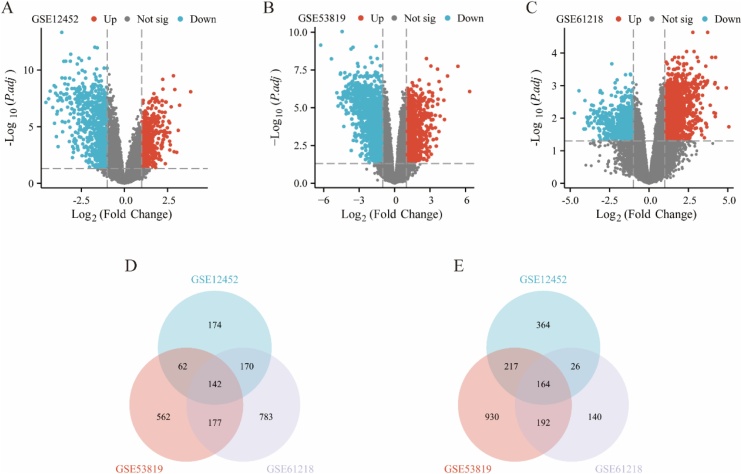
Identification of common DEGs in NPC from multiple datasets. (A‒C) Volcano plots showing the DEGs identified in three independent datasets (GSE12452, GSE53819, and GSE61218), comparing tumor tissues to normal tissues. (D) Venn diagram showing the overlapping high-expression DEGs across the three datasets. (E) Venn diagram showing the overlapping low-expression DEGs across the three datasets.

There was a total of 93 NPC specimens including 59 tumor and 34 normal tissues included in the three selected datasets (GSE12452, GSE53819 and GSE61218). GEO2R web tool was used to identify DEGs among each GEO datasets based on the criteria of adjusted *p*-value < 0.05 and |log_2_FC| > 1. A total of 1319 DEGs including 548 upregulated genes and 771 downregulated genes were identified from GSE12452, 2446 DEGs including 943 upregulated genes and 1503 downregulated genes were screened from GSE53819, and 1794 DEGs including 1272 upregulated and 522 downregulated genes were selected from the GSE61218. The volcano plots map of DEGs among each data set are shown in Fig. 1A‒C. Finally, 306 overlapping DEGs (142 high expression genes and 164 low expression genes) were identified among the three datasets using a Venn diagram (Fig. 1D‒E).

### Functional enrichment analysis of overlapping DEGs

**Fig. 2:**
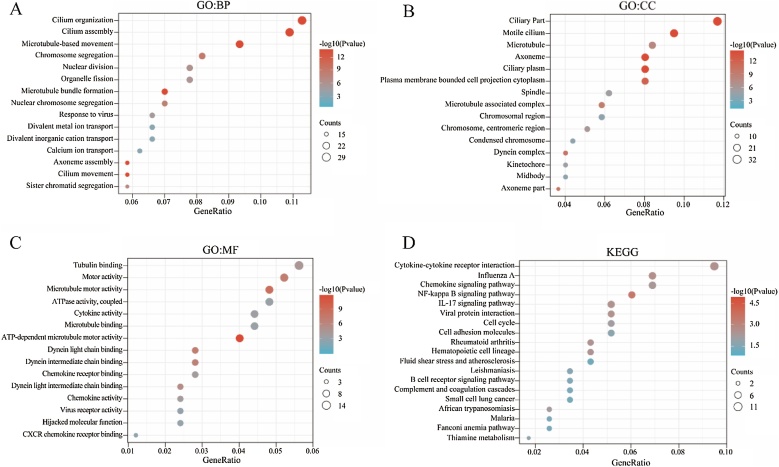
Functional enrichment analysis of overlapping DEGs. (A) GO Biological Process (BP) enrichment analysis of overlapping DEGs. (B) GO Cellular Component (CC) enrichment analysis of overlapping DEGs. (C) GO Molecular Function (MF) enrichment analysis of overlapping DEGs. (D) KEGG pathway enrichment analysis of overlapping DEGs.

To further assess the potential biological functions related to the 306 overlapping DEGs of NPC, we performed GO annotation and KEGG pathway enrichment analysis using the clusterProfiler package. In biological processes, the DEGs were mainly related to cilium organization, cilium assembly and microtubule-based movement (Fig. 2A). In cellular components, the DEGs were significantly enriched in ciliary Part, motile cilium and microtubule (Fig. 2B). In molecular function, they were mainly associated with tubulin binding, motor activity, microtubule motor activity and ATPase activity (Fig. 2C). The results of KEGG pathway enrichment analysis indicated that Cytokine-cytokine receptor interaction, Chemokine signaling pathway, NF-kappa B signaling pathway and Cell cycle were significantly enriched pathways (Fig. 2D).

### Gene set enrichment analysis (GSEA)

**Fig. 3:**
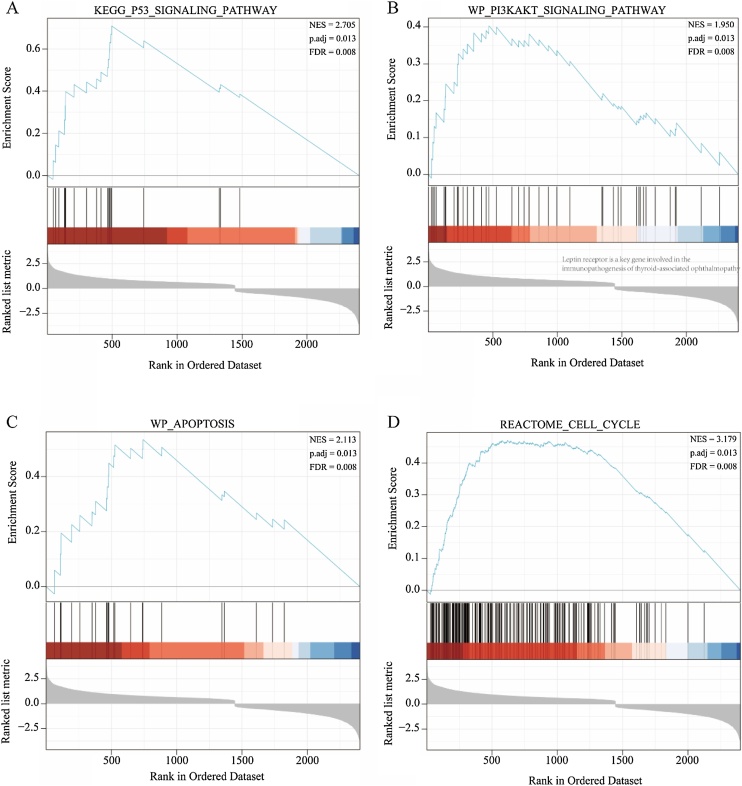
Gene Set Enrichment Analysis (GSEA) of Co-expressed Genes in NPC. (A) GSEA of the PI3K-AKT signaling pathway. (B) GSEA of the p53 signaling pathway. (C) GSEA of apoptosis-related genes. (D) GSEA of cell cycle-related genes.

GSEA was conducted to comprehensively analyze the role of all co-expressed genes from the three datasets in nasopharyngeal carcinoma. The results showed that P13K-AKT signaling pathway (Fig. 3A), P53 signaling pathway (Fig. 3B), Apoptosis (Fig. 3C) and Cell Cycle (Fig. 3D) were significantly enriched.

### PPI network construction and hub gene identification

**Fig. 4:**
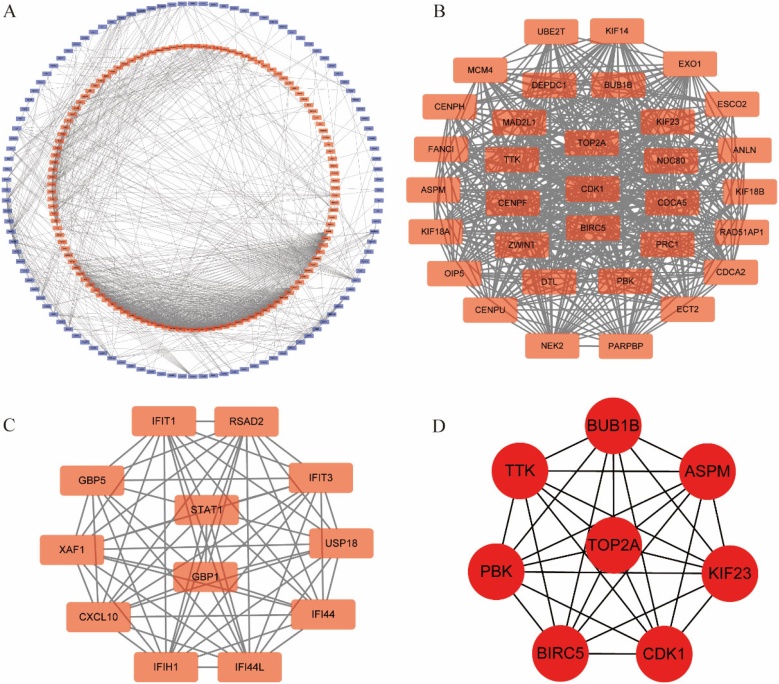
PPI Network Construction and Hub Gene Identification. (A) Protein-Protein Interaction (PPI) network of overlapping DEGs. (B) Cluster 1 extracted from the PPI network. (C) Cluster 2 identified from the PPI network. (D) Hub genes identified from the PPI network.

To explore potential correlations among proteins encoded by overlapping DEGs, a PPI network was established and visualized using the STRING database and Cytoscape software respectively. A total of 300 nodes and 1188 edges were involved in the network (Fig. 4A). To further extract vital clustering modules from the intricate network, the MCODE plug-in was applied. Subsequently, two core clusters were extracted from the PPI network according to the scores. Cluster 1 included 33 nodes and 492 edges with the highest score of 30.750 and was mainly associated with cell cycle, DNA replication, DNA Repair, chromosome segregation and apoptosis (Fig. 4B). Cluster 2 contained 12 nodes and 64 edges with a score of 11.636 and was mainly enriched in Cytokine-cytokine receptor interaction, Jak-STAT signaling pathway, Toll-like receptor signaling pathway (Fig. 4C). Finally, the cytoHubba plug-in was used to screen hub genes from the entire network, and the following top 8 genes with the highest grades were identified to be hub genes: BUB1B, CDK1, KIF23, BIRC5, TTK, TOP2A, ASPM and PBK (Fig. 4D).

### Construction of the ceRNA network

**Fig. 5:**
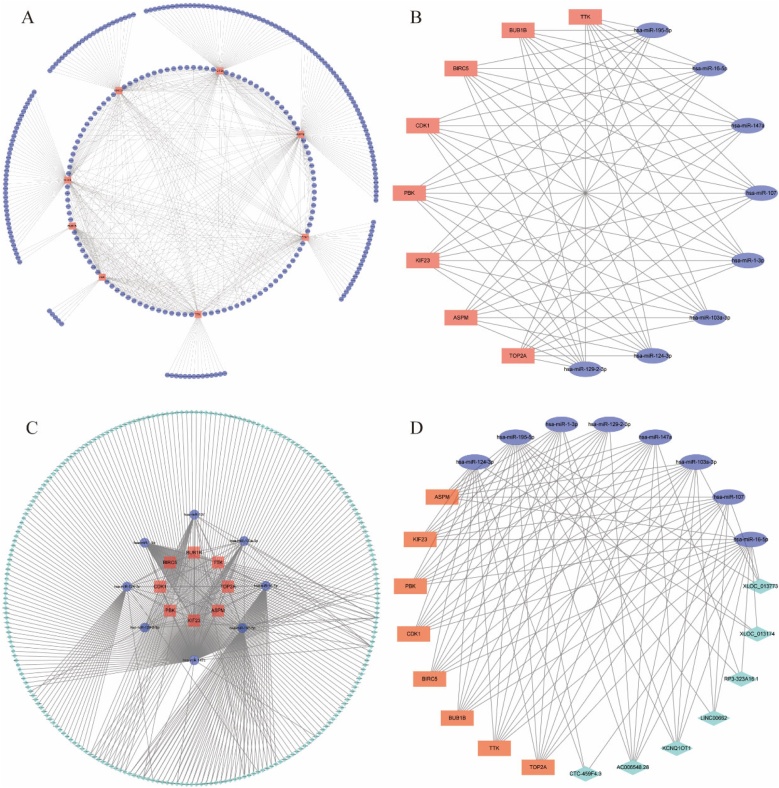
Construction of the ceRNA Regulatory Network. (A) Identification of miRNAs targeting hub mRNAs. (B) Selection of the top miRNAs with the highest connectivity. (C) Prediction of lncRNAs targeted by the selected miRNAs. (D) Construction of the ceRNA regulatory network encompassing mRNAs, miRNAs, and lncRNAs.

To establish the ceRNA regulatory network in NPC, TarBase v.8 database was firstly used to reverse identify the miRNAs upstream of hub mRNAs and a total of 540 miRNAs were found (Fig. 5A). Cytohubba plug-in then screened the top eight highly connected miRNAs (Fig. 5B). LncBase Predicted v.2 database with a threshold of 0.99 was used to predict the potential lncRNAs targeted by above 8 miRNAs and a total of 301 lncRNAs were found (Fig. 5C). Subsequently, a total of 7 lncRNAs which were both overexpressed and closely associated with the hub mRNAs were identified. Finally, a ceRNA regulatory network including 8 mRNAs, 8 miRNAs and 7 lncRNAs were constructed (Fig. 5D).

### Results of drug-gene interaction

**Fig. 6:**
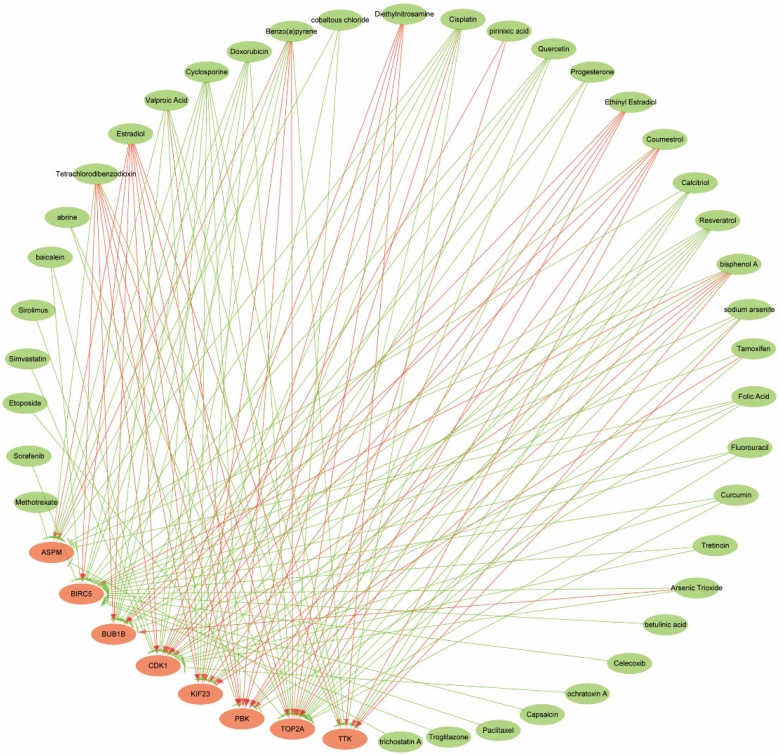
Results of Drug-Gene Interaction. The network illustrates the interactions between drugs and key genes. Green lines represent drug-mediated inhibition of key genes, while red lines indicate drug-induced activation of key genes.

The CTD database was used to explore the potential therapeutic drugs that may influence these hub genes. Various drugs including diethylnitrosamine, estradiol and tetrachlorodibenzodioxin could improve the mRNA expression level of eight hub genes, which may be risk factors for NPC. However, valproic acid, cyclosporine, doxorubicin, cisplatin, resveratrol, quercetin and calcitriol could reduce expression levels of most hub genes and may be therapeutic drugs for NPC (Fig. 6).

### Verification of hub genes and evaluation of potential therapeutic drug

**Fig. 7:**
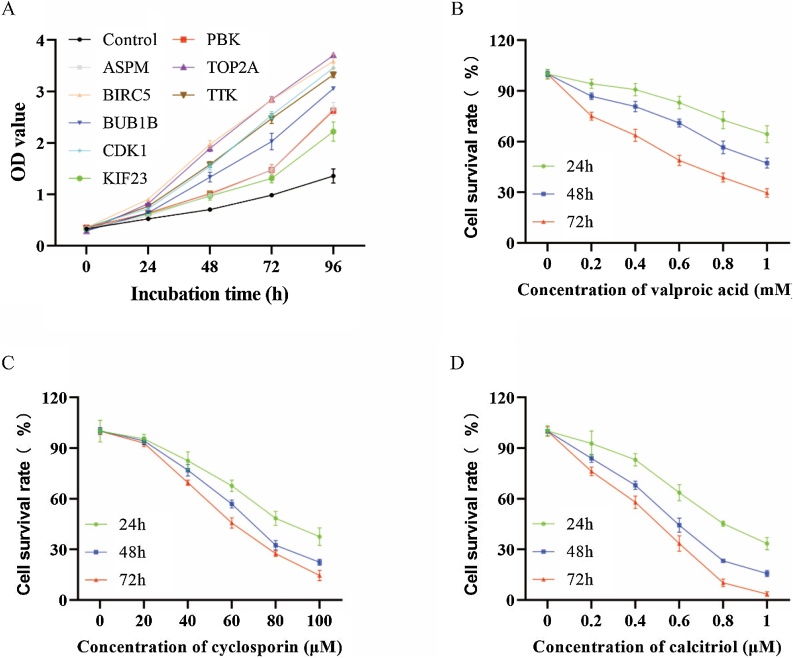
Verification of hub genes and evaluation of potential therapeutic drug. (A) CCK-8 assay assessing the effect of overexpressing the eight hub genes on NPC cell proliferation. (B) CCK-8 assay validation of valproic acid in HK1 cells. (C) CCK-8 assay validation of cyclosporine in HK1 cells. (D) CCK-8 assay validation of calcitriol in HK1 cells.

In the final part of the study, we overexpressed the eight identified hub genes in HK1 cells using plasmid transfection. The results from the CCK8 assay demonstrated that all eight genes significantly promoted the proliferation of nasopharyngeal carcinoma cells (Fig. 7A). Additionally, we tested the effects of three potential drugs ‒ valproic acid, cyclosporine, and calcitriol ‒ which have not been previously reported in NPC. The CCK-8 assay was performed to evaluate the drug effects on HK1 cells. Among the three drugs, calcitriol showed the most potent inhibitory effect on cell proliferation, indicating its potential as a promising therapeutic agent for NPC (Fig. 7B‒D). Further investigation is warranted to explore its clinical applicability.

## Discussion

Nasopharyngeal Carcinoma (NPC) is an aggressive epithelial cancer with a notably high incidence in East and Southeast Asia, particularly southern China.^26^ Despite advancements in radiotherapy and chemotherapy, the prognosis for advanced NPC remains poor due to high recurrence and metastasis rates. The molecular mechanisms driving NPC progression remain incompletely understood, emphasizing the need for further research into key genes and pathways involved in tumor development.^27^

In this study, we identified eight hub genes associated with NPC progression: ASPM, BIRC5, BUB1B, CDK1, KIF23, PBK, TOP2A, and TTK. These genes regulate key cellular processes, including cell cycle progression, DNA repair, and mitosis, and their dysregulation has been implicated in multiple cancers. For example, BIRC5 (Survivin) is a well-known anti-apoptotic protein associated with drug resistance and poor prognosis,^28^ targeted drugs such as YM155 have been tested in clinical trials for breast cancer and non-small cell lung cancer.^29^ CDK1, a crucial regulator of the G2/M transition, is frequently overexpressed in in various cancers,^30^ CDK1 inhibitors, such as RO-3306, have shown potential in inhibiting tumor cell proliferation in ovarian cancer.^31^ TOP2A is involved in DNA replication and transcription.^32^ Targeted drugs such as doxorubicin and etoposide are widely used for treating multiple cancers, including non-small cell lung cancer.^33^ TTK (MPS1) as a mitotic checkpoint kinase, is essential for cell division.^34^ TTK inhibitors, such as BAY 1161909, have demonstrated antitumor activity in clinical trials for triple-negative breast cancer.^35^ For ASPM, BUB1B, KIF23, and PBK, no approved targeted drugs are currently available. However, these genes are overexpressed in multiple cancers, suggesting their potential as therapeutic targets for future drug development.^36^, ^37^, ^38^, ^39^ In our study, the overexpression of these genes significantly promoted NPC cell proliferation, further confirming their roles in cancer progression and highlighting their potential as therapeutic targets in NPC.

Previous bioinformatics studies on nasopharyngeal carcinoma have identified key genes and pathways associated with NPC progression. For instance, WGCNA-based analysis identified 26 hub genes, including IL33 and MPP3 and SLC16A7 showing significant correlations with progression-free survival,^40^ while another study explored immune-related biomarkers FCER2, KHDRBS2, and IGSF9 as potential therapeutic targets.^41^ Other research has also screened potential NPC biomarkers, emphasizing their diagnostic and therapeutic value.^42^, ^43^ Compared to these studies, our research enhances predictive accuracy by identifying eight hub genes known for their roles in other cancers and existing targeted therapies. Moreover, we enhance the reliability of bioinformatics predictions through experimental validation, further strengthening the robustness of our findings. Additionally, our study predicted several potential therapeutic drugs through a drug-gene interaction network, including valproic acid, cyclosporine, doxorubicin, cisplatin, resveratrol, quercetin, and calcitriol. Notably, doxorubicin,^44^ cisplatin,^45^ resveratrol,^46^ and quercetin^47^ have already been shown to be effective against NPC, with some already in clinical use. Among the remaining three drugs that have not been studied in NPC ‒ valproic acid, cyclosporine, and calcitriol ‒ calcitriol demonstrated the strongest anti-proliferative effect on NPC cells, with IC50 values of 0.90 μM, 0.47 μM, and 0.31 μM at 24-, 48-, and 72-hs, respectively. This finding is particularly significant as calcitriol is a clinically approved drug with a well-established safety profile, making it an attractive candidate for repurposing in NPC therapy.

Despite these promising findings, several limitations must be addressed. Gene and drug effects were validated using only the HK1 cell line, and further studies are needed in additional NPC cell models to confirm these findings. Additionally, drug efficacy was assessed in vitro using CCK-8 assays, and in vivo validation is required to better simulate the tumor microenvironment. Lastly, clinical validation of these hub genes and therapeutic candidates in NPC patients is essential to confirm their relevance. Future studies should focus on gene expression analysis in clinical samples and conduct in vivo studies and clinical trials to evaluate calcitriol and other predicted drugs. Combining hub gene-targeting strategies with existing therapies could improve NPC treatment outcomes and help overcome drug resistance.

## Conclusion

This study identifies critical hub genes and potential therapeutic drugs for NPC, providing new insights into the molecular mechanisms underlying this cancer. The identification of calcitriol as a potential therapeutic candidate, combined with the validation of key genes, could pave the way for novel treatment strategies in NPC. This work contributes to the growing body of knowledge in NPC biology and highlights the importance of integrating bioinformatics predictions with experimental validation to advance cancer therapeutics.

## Declaration of Generative AI and AI-assisted technologies in the writing process

During the preparation of this work, the authors used ChatGPT to assist in language refinement and improve the clarity of the manuscript. After using this tool, the authors reviewed and edited the content as needed and takes full responsibility for the content of the publication.

## Funding

This work was supported by The Scientific Research Project of the Hunan Provincial Department of Education (23C0630, 24C0871, 24C0863), The Scientific research project of Hunan Provincial Health Commission (D202314029031, D202311009580), The Scientific research project of Hengyang Science and Technology Bureau (202222035595), Natural Science Foundation of Hunan Province (2025JJ80345).

## Declaration of competing interest

The authors declare that they have no known competing financial interests or personal relationships that could have appeared to influence the work reported in this paper.
